# Assessing association of household diet diversity with mother’s time use on productive and reproductive activities: a case for gender sensitive social safety nets

**DOI:** 10.1017/S1368980023002963

**Published:** 2024-01-04

**Authors:** Surabhi Chaturvedi, Sumathi Swaminathan, Sanchit Makkar, Anjaly Teresa John, Tinku Thomas

**Affiliations:** 1 Division of Epidemiology and Biostatistics, St. John’s Research Institute, Bangalore, India; 2 Division of Nutrition, St. John’s Research Institute, Bangalore, India; 3 Department of Biostatistics, St. John’s Medical College, Bangalore 560034, India

**Keywords:** Household diet diversity, Productive, Reproductive time use, Gender

## Abstract

**Objective:**

In South Asia, while women make substantial economic contributions through their participation in agricultural sector, these contributions are undercounted as most of their work is underpaid or unpaid. This paper examines how mothers allocate their time to productive and reproductive activities and its association with a household’s ability to achieve high household diet diversity score.

**Design:**

The analysis uses data on household consumption and expenditure including food during the kharif (June to October) season (seeds are sown) and a modular time-use survey.

**Setting:**

Two districts of rural Bihar, India.

**Participants:**

Mothers with children less than 5 years of age and supported by the head of the household from 2026 households.

**Results:**

The estimates indicate that the high household diet diversity (High HDDS ≥ 10) is associated with greater time spent in reproductive activities by all women (OR = 1·12, 95 % CI: 1·06, 1·18). However, with increasing time spent in productive activities by the women the odds of achieving ‘High HDDS’ reduced (OR = 0·83, 95 % CI: 0·77, 0·89) in adjusted logistic regression analysis.

**Conclusion:**

The findings highlight propensity to achieve ‘High HDDS’ in Bihar increased with mothers allocating time towards reproductive activities, while it had an opposing effect with mothers allocating time on productive activities. Our study highlights that the policies that encourage women’s participation in agriculture or livestock should acknowledge the unpaid nature of some of the productive activities and design programs to improve economic agency of women to actuate the true potential of agriculture-nutrition pathways.

At the beginning of the 21st century, various conceptual frameworks highlighted the linkages between agriculture, nutrition and health outcomes through intermediary processes of labour, gender, income, environment and multiple dimensions of food security^(1,2)^. Reviews highlighted that production of food, especially production of nutrient-rich foods was a critical factor contributing to diet diversification^(3)^. However, it was also observed that sole production of diverse foods did not ensure improved nutrition outcomes and while it led to more diverse diets at household level the extent dependent on gender and control of household decisions^(4,5)^. These and other frameworks since have emphasised the need to study the influence of women’s economic and social agency on agriculture-nutrition linkages, while also being cognisant of any potential unintended consequences such as reinforcement of gender inequities^(6,7)^.

Despite the acknowledgement of these gendered complexities, and importance given to understanding women’s work burden in the context of maternal and child health and agriculture nutrition, the evidence in the literature is scant. One known constraint has been that labour statistics often fail to capture the non-market oriented economic activities, which are largely performed by women in agrarian societies, thereby rendering them understudied^(8)^. These activities that could be unpaid and underpaid, along with unpaid care work within household can create time poverty for women, which is often overlooked or unaccounted for in nutrition, agriculture and other social protection programs^(9)^.

A systematic review on time use by women and its impact on diet diversity and nutrition outcomes found that most of the agriculture studies, which looked at the effect of agriculture-nutrition interventions on anthropometric outcomes, did not capture time use by mothers or fathers; some studied time use in context of farming activities, and not the entire gamut of activities that the mother and father might be involved in^(10)^. In India, even though women are actively involved in agricultural activities and 70 % of rural households are involved in agriculture for livelihood, there is dearth of evidence linking women’s time use with nutrition security and diets, specifically a household’s diet diversity^(11)^. Moreover, very few studies have looked at association of time use by women as an input, with aspects of nutrition security such as accessibility and affordability of nutritious food at the household level.

In this analysis, we aim to empirically understand how young mothers living in a predominantly agrarian society of rural Bihar in India allocate their time on productive (income generating or having potential to generate income but unpaid) activities or reproductive (unpaid care work – care giving) activities and how these choices have a ramification on the household diet diversity score (HDDS)^(10,12)^. It is hypothesised that increased time allocation to productive activities such as formal or informal employment opportunities could improve the income – thereby the economic agency, resulting in improved diets in the household. Additionally, increased time allocation to reproductive activities such as ‘care’ towards planning purchase and cooking with diverse nutritious foods indicates greater time expenditure or care in preparation of meals with more diverse foods and therefore could lead to better diet diversity and improved diets.

## Methods and materials

### Data source

The study was conducted in two districts (Gaya and Nalanda) in the state of Bihar between July and September, 2019, during the Kharif season (June to October), which is the pre-monsoon period when the seeds are sown. A sample of 1012 and 1014 rural households with at least one child aged 5 years or below was surveyed in Gaya and Nalanda districts respectively. The households were selected through a multi-stage cluster sampling, where villages served as a cluster (142 villages and 134 villages in Gaya and Nalanda). These districts were identified for the survey as they did not have any direct crop-based interventions going on. At the same time, these districts were involved in the production of nutrient-rich foods.

The villages were stratified based on four distance bands from the district headquarters (0–5 km, 6–15 km, 16–30 km and > 30 km) in each district to ensure representative samples with respect to proximity to urban centres and access to markets. Within a village, ten households were selected through further stratification into five categories a) landless with no food production (*n* 2), b) landholders with no nutrient-rich food production (*n* 2), c) landless involved in nutrient dense food production (*n* 2), d) small land holders with <= 2 acres involved in nutrient dense food production (*n* 3) and e) medium to large land holders with > 2 acres involved in nutrient dense food production (*n* 1). The nutrient-rich foods considered were milk, pulse, egg, chicken and green leafy vegetable. These foods were chosen as they are part of staple diet in the region, produced in this agro-ecological region and also, most likely to provide the adequate proteins and micronutrients in their diets. The selection of households within a village was done by the random walk method, where the village was split into five segments and two households were chosen from each segment.

The study was reviewed by the Institutional Ethics Committee of St. John’s Medical College, and the registration number of the study with IEC is 297/2018. We obtained written informed consent from the study participants.

### Data collection

A household questionnaire that captured socio-demographics, household consumption and expenditure (food and non-food) – adapted from household consumption and expenditure survey (HCES) of National Sample Survey was used to collect data from 2026 households. Additionally, given the context of the study, additional information on decision making on purchases within a household, household agricultural production, accessibility to markets, factors that could influence stunting of children (such as WASH indicators, household hunger), participation of women in decision-making and loans and savings was used to collect the data from 2026 households. As explained earlier, data were collected on household food and non-food consumption, expenditure and production, with special focus on nutrient-rich foods like pulses, milk, poultry, egg and green leafy vegetable.

#### Time allocation

An embedded modular time-use survey was included in the HCES to collect data on time allocation using stylised questions in the context of the larger survey^(13)^. These questions collected time-use data across six categories of activities *in an average day* by mothers of children less than 5 years of age in 2026 households. Data on time allocation were gathered towards *productive activities* such as (i) paid employment, (ii) unpaid – production of foods such as in family production/business, farm activities, livestock and fisheries and *reproductive activities* such as (iii) water collection, (iv) household work (cooking, shopping, cleaning and elderly care), (v) participation in community activities and (vi) childcare. (Mothers were asked how much time they allocated to activities under these categories.) These activities were then classified as productive (generated income or had potential to generate income, but were unpaid) and reproductive (care giving) as per the classification by Johnston and colleagues^(10)^. Specifically, productive time use was cumulative time spent on paid employment and on unpaid productive activities such as production of foods, agribusiness, engagement in livestock or fisheries, that is, time spent on activities that lead to or could lead to generation of income, yet were unpaid and reproductive time use was time spent on household work, water collection and childcare and unpaid community engagement^(14)^.

Household Diet Diversity: The HDDS is meant to reflect, in a snapshot form, the economic ability of a household to access a variety of foods. Studies have shown that an increase in dietary diversity is associated with socio-economic status and household food security (household energy availability)^(15)^. This score was calculated using twelve food groups consumed in the previous 7 d by the household. The survey recorded household food consumption and expenditure and frequency. Data on the kinds and amounts of food consumed by the household were collected using a food list and other memory aids (such as store receipts and menus). The food groups considered the recall period for each of the food groups and the method of extraction of data are presented in online supplementary material, Supplemental Table 1. The HCES format survey collected household consumption and expenditure using the typical recall period of 30 d. Barring food items falling under carbohydrates, sugar and oil, the recall period for all the other food items in other food groups was also collected using a past-7 d recall. (Since carbohydrates, sugar and oil are staple food items consumed every day in the region.) Thus, we obtained a HDDS score which is a count of food groups consumed in the last week, out of the twelve food groups listed in online supplementary material, Supplemental Table 1. The scores therefore range between 0 and 12. A household was considered to achieve minimum threshold for nutrient adequacy if the HDDS score was >= 10^(16)^. As per Mekonnen et al., this is the minimum threshold at which HDDS may improve household’s mean probability of nutrient adequacy (MPA). While this score does not estimate the MPA, using this rationale, HDDS was categorised into two: 1. ‘High HDDS’ (Score 10–12) and 2. ‘Low HDDS’ (Score 0–9), a binary dependant variable.

### Statistical methods

All categorical data were summarised as numbers (count) and percentages. After checking for normality, continuous data that were not normally distributed are presented as median and quartiles. The association of HDDS (the binary variable) with productive and reproductive time use of mothers with children < 5 years of age was examined separately, using binary logistic regression models and the results reported as unadjusted and adjusted OR with 95 % CI (95 % CI). The association of HDDS with time use was also examined with polynomial logistic regression to allow time use to be nonlinear. Household characteristics that included data on household production diversity (whether the household produced nutrient-rich foods such as dairy, pulses, green leafy vegetables, poultry, egg or not – a dichotomous variable), household food expenditure share in total expenditure (proxy for income – income quartile based on total household expenditure spent on food: Engel’s Law. (I1-least percentage – I4-highest percentage)), household size and gender, education and age of the household head were considered as confounders in the model. Involvement of women in decision making regarding purchase and involvement of women in household production of nutrient-rich foods were also included in the models. The choice of confounders was based on existing literature and variables captured in the study^(17–20)^.

All data analysis was performed using STATA version 13 (StataCorp. 2013. Stata Statistical Software: Release 13.).

## Results

The total number of women who responded was 2026. The characteristics of the surveyed households are presented in Table 1. Majority of the household heads were male (87·8 %) and 56 % of the households owned agricultural land (of which 92 % were marginal farmers). About 47 % of the households reported agriculture to be the primary occupation. Median household monthly expenditure on food for agricultural household was INR 6952 (Q1:5004, Q3: 9386), as opposed to INR 4968 (Q1:3696, Q3: 7075) for non-agricultural households. However, the monthly expenditure on food for households that engaged in casual labour was substantially lower than others in for both agricultural and non-agricultural households (INR 4881 (Q1:3419, Q3:7347) and INR 4636 (Q1:3454, Q3: 6303), respectively). Overall, 64 % of the households were involved in the production of any of the five nutrient-rich foods. 51 % of women reported allocating time on productive activities and all women allocated time towards reproductive activities.

**Table 1:** Household socio-demographic characteristics

Characteristics	*n*	Percentages (%)	
Sex of head of household			
Male	1778	87·8	
Household head – education			
Illiterate	788	38·91	
Primary or less	442	21·83	
High school or less	496	24·49	
Completed higher secondary	155	7·65	
Diploma, graduate or more	144	7·11	
Primary occupation			
Agriculture			
Self-employed	892	44·03	
Casual labourer	67	3·31	
Non-agriculture			
Self-employed	215	10·61	
Casual labourer	469	23·15	
Regular wage/Salaried	383	18·9	
Agriculture land owned	1142	56·4	
Land holding size by agriculture land owner (%)			
Marginal (0 < 2·5 acres)	1050	91·9	
Small (2·5–4·9 acres)	70	6·1	
Medium to large (5–30 acres)	22	1·9	
Household produces nutrient-rich foods	1294	63·9	
Women in household involved in production of nutrient-rich foods			
Milk	397	19·6	
Green leafy vegetables	191	9·4	
Pulses	353	17·4	
Chicken	31	1·5	
Egg	35	1·7	
Household by type of diet			
Vegetarian	93	4·59	
Eggetarian	5	0·25	
Non-vegetarian	1928	95·16	
Proportion of women – time-use allocation	2026		
Productive work	1039	51 %	
Reproductive – unpaid work	2026	100 %	
Women in the household involved in decision making to purchase nutrient-rich foods			
Milk	712	35·1	
Green leafy vegetables	371	18·3	
Pulses	851	42	
Chicken	433	21·4	
Egg	287	14·2	
Continuous variables	*n*	Median	Quartile1, Quartile3
HH size (number)	2026	6	5, 8
Age of HH head (years)	2024	40	30, 55
Household expenditure on food if agriculture is primary occupation (Indian Rupees*)	959	6952	5004, 9386
Self-employed	892	7061	5167, 9654
Casual labourer	67	4881	3419, 7347
Household expenditure on food if non-agriculture is primary occupation (Indian Rupees*)	1067	4968	3696, 7075
Self-employed	215	5354	4112, 7407
Casual labourer	469	4636	3454, 6303
Regular wage/salaried	383	5347	3666, 7888

* 1 USD = 71·03 INR as of November 2019.

The mean HDDS was 9 (sd = 1·3) and the HDDS ranged from 5 to 12. ‘High HDDS’ (HDDS ≥ 10) was reported by 24 % of the households. The lower HDDS was mainly due to the non-consumption of non-vegetarian foods (fish/seafood-96 %, eggs-86 % and meat/poultry-40 %) although 95 % of them reported that they were non-vegetarian.

In the sample of mothers interviewed for time use, 7 % of women allocated time on both paid *and* unpaid productive work, 8 % of women were involved in paid productive activities only and 37 % in unpaid productive activities only (Table 2). Given the small sub-sample of women doing paid productive work, the study was underpowered to examine the association in a group of women with paid work alone. Therefore, a cumulative productive time use (including 51 % of women) and reproductive time-use categorisation was used for the purpose of this analysis.

**Table 2:** Time use of women with children < 5 years of age

	*n*	Median	Quartile 1, Quartile 3
Productive time use (minutes)	2026	20	0, 120
Productive time use (minutes) for those involved in productive activities	1039	120	60, 240
Paid work only	159	300	180, 480
Unpaid work only	741	110	60, 120
Paid and unpaid	139	420	270, 540
Reproductive time use (in minutes)	2026	380	310, 480
Water collection	1188	10	5, 30
Household chores	2009	180	120, 240
Childcare	2017	158	120, 240
Community-unpaid work	559	60	60, 61

Productive unpaid work includes time allocated for production of household food production. The N for sub-categories of reproductive work does not add upto 2026 as women allocated time towards multiple sub-categories of water collection, household chores, childcare and community-unpaid work.

Median productive time use for all women (Table 2) was 20 min/d (Q1:0 min, Q3:120 min). Among the women who reported allocating time towards productive activities (51 %), the median productive time use was 120 min/d (Q1: 60, Q3: 240). Productive time use was highest for women who engaged in both paid and unpaid productive activities (Median time = 420 min), compared with women who allocated time towards paid productive activities only (Median time = 300 min) and unpaid productive activities only (Median time = 110 min). All the women reported participating in reproductive activities and the median reproductive time use was 380 min/day (Q1:310 min, Q3:480 min). Among reproductive activities, the maximum time was for household work (Median time = 180 min) followed by childcare (Median time = 158 min).

**Fig. 1:**
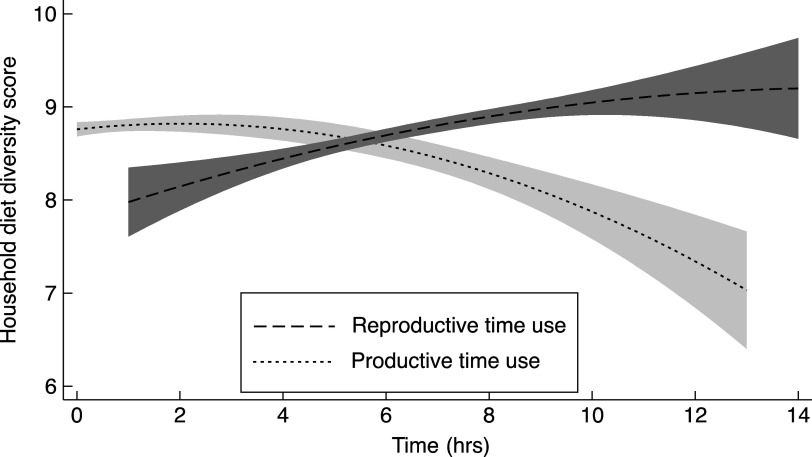
Association of HDDS with reproductive time use and productive time use. HDDS, household diet diversity score.

There was a negative association of productive time use with High HDDS and positive association with reproductive time use (Fig. 1). The association of time use and achieving HDDS (HDDS ≥ 10) is reported in Table 3. The odds of High HDDS increased with greater time spent in reproductive activities by all women (OR = 1·12, 95 % CI: 1·06, 1·18). However the odds of ‘High HDDS’ were lower with increasing time spent on productive activities for all women (OR = 0·83, 95 % CI: 0·77, 0·89). The nonlinear association of productive time use was significant with the first-order term being positive (OR = 3·36, 95 % CI: 1·19, 9·49) and the second-order term being negative (OR = 0·51, 95 % CI: 0·33, 0·80). However, the correct classification based on the nonlinear regression improved by only 1 % (77 % *v*. 76 %) compared with the linear term model and so the second-order term can be ignored.

**Table 3:** Association of adequate diet diversity with women’s time use and household characteristics

	Productive time-use model			Reproductive time-use model		
Dependent variable: Adequate household diet diversity (HDDS 10–12) – high HDDS	OR	95 % CI	*P* value	OR	95 % CI	*P* value
Time use (Hours)	0·83	0·77, 0·89	< 0·001	1·12	1·06, 1·18	< 0·001
Involved in productive activities (No)	0·47	0·35, 0·62	< 0·001			
Income quartile (Ref: First)						
Second	1·29	0·94, 1·78	0·10	1·32	0·97, 1·81	0·08
Third	1·45	1·06, 1·98	0·02	1·56	1·14, 2·12	0·01
Forth	1·19	0·86, 1·67	0·29	1·41	1·02, 1·96	0·04
Household involved in production of nutrient-rich food (Yes)	1·22	0·95, 1·57	0·12	1·31	1·02, 1·68	0·03
Household head’s education (Ref: Illiterate)						
Primary or some level of schooling	1·37	1·02, 1·83	0·04	1·37	1·02, 1·84	0·04
High school and middle school	1·31	0·98, 1·75	0·07	1·33	1·02, 1·84	0·06
Higher Secondary	1·26	0·82, 1·96	0·28	1·37	0·89, 2·11	0·15
Diploma, Graduate	1·62	1·06, 2·50	0·03	1·69	1·10, 2·60	0·02
Household size	1·05	1·00, 1·09	0·04	1·04	0·99, 1·09	0·08
Household head’s gender (Female)	0·50	0·32, 0·72	< 0·001	0·49	0·33, 0·72	< 0·001
Women in HH involved in decision making to purchase nutrient-rich foods						
Green leafy vegetables	1·54	1·17, 2·03	< 0·001	1·58	1·20, 2·07	< 0·001
Chicken	1·57	1·17, 2·12	< 0·001	1·54	1·15, 2·07	< 0·001
Egg	2·73	1·96, 3·78	< 0·001	2·67	1·94, 3·69	< 0·001
Milk	1·29	1·01, 1·67	0·04	1·21	0·95, 1·55	0·12
Women in HH involved in production of nutrient rich foods						
Egg	2·43	1·00, 5·88	0·05	2·65	1·11, 6·32	0·03
Milk	1·20	0·90, 1·61	0·20	1·22	0·91, 1·62	0·18
Chicken	1·77	0·69, 4·50	0·23	1·89	0·75, 4·79	0·18

HDDS, household diet diversity score.

OR: Adjusted OR using binary logistic regression with all variables in the table as independent variables.

Based on the purposeful selection of covariates in bivariate analysis – women involved in production of pulses and green leafy vegetable, purchase-decision making of pulses and household head’s age were not included in the models. (These covariates that were NS at *P* < 0·2 were not considered for further analysis. see online supplementary material, Supplemental Table 2.) All models were adjusted for household size, income of the household and gender, education of the household head and if the household was involved in production of nutrient rich foods. Households with women involved in production of milk and chicken were not associated with High HDDS; however, households that had women involved in production of eggs were significantly associated with odds of High HDDS in all the models. Women’s involvement in decision making about purchase of green-leafy vegetables, egg, chicken and milk was also positively associated with High HDDS in the productive time-use model (full and adjusted model); the decision making about purchase of milk was NS in the reproductive time-use model.

Household income and household size were not associated with women’s involvement with productive activity (see online supplementary material, Supplemental Table 3). In a subgroup (*n* 1039) of women who engaged in productive activities, the odds of High HDDS continued to be lower (OR = 0·82, 95 % CI: 0·76, 0·88) with increasing time spent in productive activities (see online supplementary material, Supplemental Table 4), based on an adjusted model. In the subgroup (*n* 987), women who did not allocate any time in productive activities and only spent time spent in reproductive activities the odds of achieving High HDDS was not associated with time on these reproductive activities (see online supplementary material, Supplemental Table 4).

## Discussion

The paper assessed the association of women’s labour, specifically for mothers with young children, measured in terms of productive and reproductive time use and its association with a household’s minimum probability to achieve High HDDS. This was also an attempt to use a less expensive modular time-use survey as part of a larger household consumption, expenditure and food production survey, to shed light on time use as a labour statistic to highlight women’s contribution towards economic activity.

The analysis in this paper finds that on one hand, there is significant positive relationship between increased time on reproductive activities and improved odds of households achieving household diet diversity ≥ 10, which corresponded to achieving minimum nutrient adequacy in Ethiopia. However, increased time allocation towards productive activities by mother of young children leads to a decreased probability for a achieving this high household diet diversity score. The former is in alignment with the evidence on meal preparation and food security, but the latter is counter-intuitive at first^(17)^. The former could be explained by looking at the break-up of time use on reproductive activities. All the young mothers in the sample reported spending maximum time on household chores, which included cooking and shopping, amongst other activities. This could be interpreted as more time expenditure in planning, and preparation of nutritious meals, which translates into improvement of nutrient adequacy and access dimension of food security for the household, as expected in the hypothesis for this analysis.

For productive time-use’s association with HDDS, theoretically as per the agriculture-gendered linkages, access to income in return for her labour and agency in decision making should allow women to purchase more nutritious diets and should have a positive impact on a household’s nutrition security. However, our sample shows that only a little over half of the young mothers reported participating in any productive activities, out of which most reported engaging in farm activities, fisheries and livestock in family-owned or local businesses (43 %) which were unpaid. These activities in India are conventionally unpaid, and at best underpaid – a proxy for unrealised income^(21)^. Very few women reported working in paid employment opportunities. The issue of invisible unpaid contribution by women could be the reason for a significant negative association on HDDS.

The findings add to the existing evidence by that used time-use methodology to study nutrient intake, women and children’s diet diversity. Vemireddy and colleagues used a panel data from India and found that working extended hours in agriculture was associated with a reduction in women’s nutrient intake in terms of calories, proteins, fats, Fe and Zn^(22)^. Komatsu and colleagues, using data on time use by women and diet diversity from four developing countries observed that, in some countries, when women worked long hours in agriculture-based activities, a negative relationship was observed between work in agriculture and women and children’s diet diversity^(23)^. However, there were no consistent benefits on women’s and children’s diet diversity and the impact differed based on poverty levels of the household and country context. Sangwan and colleagues found that women’s participation in labour force had a positive impact on household’s dietary diversity; however, there was significant heterogeneity in this effect by type of work – paid and unpaid labour^(24)^.

This finding is crucial because it highlights that perhaps even if women are participating in productive and economic activities, in absence of adequate remuneration, this only increases their workload^(25)^. Due to unpaid nature of these roles, not only is their economic contribution rendered invisible exacerbating their pre-existing economic marginalisation in absence of land rights but also it hampers a household’s economic ability to access diverse and nutrient adequate diets^(26,27)^.

### Limitations and areas of further research

Given the modular nature of the time-use survey, we were unable to glean the exact nature of these productive activities (formal or informal paid employment opportunities and productive activities which are home based or closer to home or on-farm roles). Moreover, given only 7 % of women allocated time towards with both paid and unpaid productive activities, the sub-sample was underpowered to examine the association in sub-groups of women with paid and unpaid productive work separately. Our survey also did not collect data on the wages earned by young mothers who were engaged in ‘paid employment’ activities, a sub-category of productive time use. It is interesting to note that while we did not collect the time use for the other women in the household, we adjusted the model for households where women participated in purchasing decision and production of nutrient-rich foods. Women’s involvement in production of eggs and participation in purchasing decisions for egg, chicken, and green leafy vegetables were significant and positively associated with a household’s economic access to a diverse diets. Furthermore, in future research, it might be pertinent to not only focus on intra-household dynamics within a household but also focus on intra-women’s dynamics within the household, accounting for their age, time use and nature of productive activities they participate in to see its effect on nutrition security and to also capture if these dynamics compromise a young women’s earning potential.

Furthermore, given the inherent limitation of a short modular time-use survey, it is possible that we might have missed time allocation on some region-specific activities that could have been captured through data collected from time diaries. Hence, when using modular time-use surveys, it might be useful to customise and contextualise the time-use modules through pre-testing to ascertain the extent of detailing of the time-use module. Moreover, and this might require more detailed time-use modules, and may not be possible in modular time use survey, there is a need to capture the exclusive time use on childcare. Time spent on one activity comes at the expense of the other. There must be time trade-offs between time towards productive activities and reproductive activities. However, since a modular time-use survey was used, which asks stylised questions related to main survey, the collected survey does not capture time allocation comprehensively, hence is not adequate to assess time-trade-off. Future research should further focus on time-trade-offs in productive and reproductive activities, and the nature of the work, to also understand the trade-offs effect on child growth and development outcomes. While the module used in this analysis had a separate category on childcare, it is a concern that childcare in these household might be a secondary activity or more custodial in nature instead of the nurturing care that actually benefits child development^(28–31)^. Therefore, even though women in our sample reported spending a median of 150 min on childcare, there would be a need to further investigate the quality of this care.

Moreover, in this context, it would be important that future surveys and impact studies also explore the linkages across women’s time use, household’s food security and potential unintended consequences on women’s own well-being and children’s nutrition and development, before extrapolating the results that increased time in reproductive activities by the mother is good for household’s access and consumption of nutrient-rich foods^(32–35)^.

Furthermore, time allocation by women between productive and reproductive activities might vary based on agriculture season and regions under study. While our findings pertain to the Kharif season one round of survey, to account for the above, and capture seasonality of production and consumption, data have been collected for the Rabi season as well, which might shed more light on the complexities of intra-household dynamics, role of women and agriculture and its subsequent impact on nutrition security^(36–38)^.

### Conclusion

While there is a opposing effect in our analysis, of women’s time use on productive and reproductive activities and High HDDS, this highlights the need to better understand the nature of productive activities, unpaid work done by women and, subsequently, the magnitude of unrealised income from this labour and gender linkages. Policies that influence level of wages/entrepreneurial income, labour regulations and stipulations, availability of formal-informal jobs, enabling social protection services such as childcare and societal empowerment of women in decision making on income allocation for food will have an impact on nutrition outcomes, especially child development, and various aspects of nutrition security.

## Supporting information

Chaturvedi et al. supplementary material Chaturvedi et al. supplementary material

## Supplementary material

For supplementary material accompanying this paper visit https://doi.org/10.1017/S1368980023002963
