# Supplementary material for: Assessing association of household diet diversity with mother’s time use on productive and reproductive activities: a case for gender sensitive social safety nets
Source: Public Health Nutr. 2024 Jan 4;27(1):e33. doi: 10.1017/S1368980023002963 (PMC10897577; doi:10.1017/S1368980023002963)
Supplement: Chaturvedi et al. supplementary material [file S1368980023002963sup001.docx]

***Supplementary Material***

***Table 1: Construction of Household Diet Diversity Score using different food groups and associated recall period***

|  | **Food Groups** | **Recall Period** |
| --- | --- | --- |
| 1 | Cereals | Monthly (Cereals are a staple part of diet in India and consumed every day, hence this was assumed to be consumed weekly) |
| 2 | Roots & tubers | Weekly & Monthly |
| 3 | Vegetables (Green leady vegetables and other vegetables, excluding roots and tuber) | Weekly & Monthly |
| 4 | Fruits | Weekly & Monthly |
| 5 | Meat, and poultry | Weekly & Monthly |
| 6 | Eggs | Weekly & Monthly |
| 7 | Fish and seafood | Weekly & Monthly |
| 8 | Pulses, legumes and nuts | Weekly & Monthly |
| 9 | Milk and milk products | Weekly & Monthly |
| 10 | Oils/Fats | Monthly (Oils are used for cooking and consumed every day in India, hence this was assumed to be consumed weekly) |
| 11 | Sugar/honey | Monthly (Sugar is consumed daily with tea and milk in India, hence this was assumed to be consumed weekly) |
| 12 | Miscellaneous (Beverages & Other Processed Foods) | Weekly |

***Supplementary Table 2: Purposeful selection of covariates in the full model***

| HDDS_binary | OR (95% Confidence interval) | p-value |
| --- | --- | --- |
| Households involved in production of nutrient rich foods | 1.41 | 0.00 |
| Household head’s gender | 0.62 | 0.01 |
| Household women involved in production of pulses | 0.93 | 0.62 |
| Household women involved in production of green leafy vegetables | 0.99 | 0.96 |
| Household women involved in production of chicken | 3.52 | 0.01 |
| Household women involved in production of eggs | 3.11 | 0.01 |
| Household women involved in production of milk | 1.33 | 0.03 |
| Household women involved in decision making for purchase of pulses | 1.05 | 0.64 |
| Household women involved in decision making for purchase of green leady vegetables | 1.89 | 0.00 |
| Household women involved in decision making for purchase of chicken | 2.11 | 0.00 |
| Household women involved in decision making for purchase of eggs | 3.29 | 0.00 |
| Household women involved in decision making for purchase of milk | 1.32 | 0.01 |

***Supplementary Table 3: Sub-group analysis - Women’s involvement in productive time-use activity – Household Size and Income Quartile***

| **Descriptive Statistics for sub-group** | | | |
| --- | --- | --- | --- |
|  | **Women with productive-time use**  **(N=1039)** | **Women without productive-time use (Reproductive time use only)**  **(N=987)** |  |
| **Household Size Median (Quartile 1,Quartile 3)** | 6(5,8) | 6 (5,8) |  |
| **Income Quartile** | Percentages (%) | Percentages (%) |  |
| I1 | 21.6% | 28.7% |  |
| I2 | 23.1% | 27.0% |  |
| I3 | 26.7% | 23.3% |  |
| I4 | 28.7% | 21.1% |  |
| ***Association with women’s involvement in productive time-use activity – Household Size and Income Quartile*** | | | |
| **Dependant Variable: Women engaged in productive activity (Binary variable) – N=2026** | | | P Value |
| **Household Size** | 1.02 (0.99-1.06) | | 0.15 |
| **Income Quartile (Ref: First)** |  | |  |
| Second | 1.14 (0.89-1.46) | | 0.29 |
| Third | 1.52 (1.19-1.95) | | 0.001 |
| Forth | 1.8 (1.41-2.32) | | 0.000 |

**Supplementary Table 4: Association of adequate diet diversity with women’s time use for women who engage in productive time-use and for those who do not and household characteristics**

| **Dependent Variable: Adequate household**  **diet diversity (HDDS 10-12) – High HDDS** | Women with Productive time-use (n=1039) | P Value | Women without Productive time-use (Reproductive time-use only) (n=987) | P Value |
| --- | --- | --- | --- | --- |
|  | OR (95% Confidence interval) |  | OR (95% Confidence interval) |  |
| **Productive Time-use (Hours)** | 0.82 (0.76-0.88) | 0.00 |  |  |
| **Reproductive Time-use (Hours)** |  |  | 1.01 (0.93-1.11) | 0.75 |
| **Income Quartile (Ref: First)** |  |  |  |  |
| Second | 1.64 (1.02-2.61) | 0.04 | 1.05 (0.68-1.63) | 0.83 |
| Third | 1.74 (1.1-2.74) | 0.02 | 1.22 (0.78-1.92) | 0.39 |
| Forth | 1.27 (0.78-2.05) | 0.32 | 1.29 (0.79-2.11) | 0.29 |
| **Household involved in production of nutrient rich food** (Yes) | 1.25 (0.86-1.79) | 0.229 | 1.25 (0.86 - 1.80) | 0.25 |
| **Household head's education**  **(Ref: Illiterate)** |  |  |  |  |
| Primary or some level of schooling | 1.38 (0.92-2.05) | 0.11 | 1.44 (0.91-2.27) | 0.12 |
| High school and middle school | 1.36 (0.91-2.03) | 0.13 | 1.25 (0.81-1.93) | 0.32 |
| Higher Secondary | 0.57 (0.28-1.17) | 0.13 | 2.23 (1.25 -3.95) | 0.01 |
| Diploma, Graduate | 1.8 (0.99-3.27) | 0.05 | 1.48 (0.78 -2.79) | 0.23 |
| **Household Size** | 1.08 (1.02-1.15) | 0.01 | 1.00 (0.94-1.07) | 0.93 |
| **Household head's gender (Female)** | 0.33 (0.18-0.61) | 0.00 | 0.64 (0.37-1.10) | 0.11 |
| **Women in HH involved in decision making to purchase nutrient rich foods** |  |  |  |  |
| Green leafy vegetables | 1.95 (1.32-2.88) | 0.001 | 1.26 (0.84-1.88) | 0.26 |
| Chicken | 1.56 (1.02-2.39) | 0.04 | 1.67 (1.07-2.58) | 0.02 |
| Egg | 2.4 (1.48-3.9) | 0.00 | 3.33 (2.08-5.26) | 0.00 |
| Milk | 1.39 (0.97-2.00) | 0.08 | 1.13 (0.79-1.62) | 0.49 |
| **Women in HH involved in production of nutrient rich foods** |  |  |  |  |
| Egg | 3.15 (1.14-8.69) | 0.03 | 1.45 (0.15-14.25) | 0.75 |
| Milk | 0.96 (0.65-1.4) | 0.82 | 1.87 (1.18-2.98) | 0.01 |
| Chicken | 2.24 (0.77-6.57) | 0.14 | 1.19 (0.11-12.42) | 0.89 |
